# Enhanced recovery after surgery in primary liver cancer patients undergoing hepatectomy: experience from a large tertiary hospital in China

**DOI:** 10.1186/s12893-023-02040-4

**Published:** 2023-06-29

**Authors:** Huan Huang, Ping Zhou, Jia Li, Hongping Luo, Liping Yu

**Affiliations:** 1 Center for Nurturing Care Research, School of Nursing, Wuhan University, Wuhan, 430071 China; 2 Departement of hepatic surgery, Tongji Hospital, Huazhong University of Science and Technology, Wuhan, 430030 China

**Keywords:** ERAS, Liver cancer, Hepatectomy, Complication, Hospitalization stay

## Abstract

**Objective:**

Enhanced recovery after surgery (ERAS) has significant effects in gastrointestinal surgery, urology, and orthopedic department, but the application of ERAS in liver cancer patients undergoing hepatectomy is less reported. This study aims to identify the effectiveness and safety of ERAS in liver cancer patients undergoing hepatectomy.

**Methods:**

Patients who performed ERAS and no-ERAS after hepatectomy due to liver cancer from 2019 to 2022 were prospectively and retrospectively collected, respectively. Preoperative baseline data, surgical characteristics, and postoperative outcomes of patients in ERAS and non-ERAS groups were compared and analyzed. Logistic regression analysis was conducted to identify the risk factors of complications occurrence and prolonged hospital stay.

**Results:**

In total, 318 patients were included in the study, 150 and 168 individuals in the ERAS group and non-ERAS group, respectively. The preoperative baseline and surgical characteristics between the ERAS and non-ERAS groups were comparable and not statistically different. Postoperative visual analogue scale pain score, the median day of gastrointestinal function recovery postoperative, complications rate, and postoperative hospitalization days were lower in the ERAS group than in the non-ERAS group. In addition, multivariate logistic regression analysis found that the implementation of the ERAS was an independent protective factor for prolonged hospitalization stay and complications occurrence. The rate of rehospitalization after discharge (< 30 days) in the ERAS group was lower than that in the non-ERAS group, but there was no statistical difference between the two groups.

**Conclusions:**

The application of ERAS in hepatectomy for patients with liver cancer is safe and effective. **I**t can accelerate gastrointestinal function recovery postoperative, shorten the length of hospital stay, and reduce postoperative pain and complications.

**Supplementary Information:**

The online version contains supplementary material available at 10.1186/s12893-023-02040-4.

## Introduction

Based on the 2022 updated data of China, primary liver cancer is the fourth cancer type and the second leading cause of cancer death, which seriously threatens the survival outcome of patients [1, 2]. Hepatectomy is an important treatment for the long-term survival of patients with primary liver cancer [3]. With the rapid development of surgical techniques and equipment, laparoscopic hepatectomy has the advantages of less trauma and faster postoperative recovery [4, 5], but the severe and distressing postoperative complications still plague patients [6, 7]. Common complaints manifest as pleural effusion, peritoneal effusion, bile leakage, organ function failure, deep vein thrombosis, postoperative hemorrhage, pulmonary embolism, surgical site infection, and so on [7, 8], which can prolong the length of hospital stay and increase the hospital cost.

The concept of enhanced recovery after surgery (ERAS) was first introduced by H. Khelet in 1997 [9]. The main purpose of ERAS is to reduce the rate of postoperative complications and to shorten hospital stays. ERAS has been reported to be effective in reducing moderate or severe complications and shortening the length of hospital stay in patients undergoing colonic resection [10]. Furthermore, ERAS has been successfully applied to urological [11], gynecological [12], orthopedic [13], and cardiac surgery [14]. Since the publication of the first ERAS guideline for liver surgery in 2016, ERAS has been widely used in liver surgery, and pieces of evidence also show that ERAS can improve the postoperative outcome of liver surgery [15]. In 2022, the latest ERAS guideline for liver surgery was released, which contains 25 recommendations. Based on the 2016 ERAS guideline, three new recommendations were added, including prehabilitation, preoperative biliary drainage, and preoperative smoking and alcohol withdrawal, and other items were reevaluated to increase the clinical applicability of ERAS [16, 17]. Currently, the literature on ERAS after hepatectomy for patients with liver cancer is still limited and some studies had a small sample size. The purpose of this study is to further evaluate the effect of ERAS after hepatectomy for primary liver cancer patients.

## Methods

### Participants

This study enrolled hepatectomy patients diagnosed with liver cancer from January 2019 to March 2022 in Tongji Hospital of Huazhong University of Science and Technology. Data from patients who performed ERAS were prospectively collected and data from no-ERAS patients were collected retrospectively. All procedures of this study were performed in accordance with the Declaration of Helsinki and the approval arrangement of the Ethics Committee of the Tongji Hospital of Huazhong University of Science and Technology, and the signed informed consent approved from all participants was received.

Patients who met the following inclusion criteria were enrolled in the study: ①: adults (aged ≥ 18) who were diagnosed with primary liver cancer without distant metastasis diseases; ②: Curative hepatectomy for the first time due to primary hepatocellular carcinoma (intrahepatic cholangiocarcinoma and combined hepatocellular-cholangiocarcinoma were excluded); ③: The liver function was Child-Pugh grade A and indocyanine green retention rate at 15 min (ICG-R15) < 30%; ④: All patients signed informed consent for curative hepatectomy.

Patients who were not complete the entire ERAS protocol or gave up halfway, and without follow-up for more than 30 days for both the ERAS group and non-ERAS group were not included.

The ERAS protocol used in this study was developed based on the latest recommendations of the ERAS Association for liver surgery [17] and the nursing characteristics of our center. Details were summarized in Supplementary Table 1.

### Study variables

Data included the demographic characteristics of participants (age at diagnosis, gender, educational level, Body Mass Index (BMI), comorbidity, chronic smoking and drinking history) and the information related to clinical characteristics (American Society of Anesthesiologists grade [ASA], glutamic pyruvic transaminase, glutamic oxaloacetic transaminase, platelet, hemoglobin, white blood cell, albumin, total bilirubin, surgical approach, extent of surgically removed liver segments, surgical time, and intraoperative blood loss). Complications after hepatectomy include nausea, vomiting, abdominal distension, wound infection, pleural effusion, abdominal effusion, hepatic encephalopathy, venous thrombosis of the lower limbs, liver failure, hemorrhage, and bile leakage. The length of postoperative hospital stay was defined as the day of hepatectomy to the day of discharge. We evaluated the patient’s postoperative visual analogue scale pain score on the 1, 2, and 3 days postoperative. Gastrointestinal function recovery was defined as the first bowel movement after surgery.

### Study outcome

The primary outcome measures were: ①: postoperative pain score; ②: the median day of gastrointestinal function recovery postoperative; ③: postoperative complications rate; ④: postoperative hospitalization days; ⑤: total hospital cost; and ⑥: rehospitalization after discharge (< 30 days).

### Statistical analysis

Categorical variables were presented as frequencies (%) and were compared using chi-square test. Continuous variables were described as the mean (standard deviation [SD]), and if they have a normal distribution compared with a student’s t-test or as the median (interquartile range [IQR]) if they did not have a normal distribution compared with a Wilcoxon rank-sum test. Univariate and multivariate Logistic regression analyses were conducted to find the impact factors of complications occurrence and prolonged hospital stay. A p-value < 0.05 was considered to represent a statistically significant difference, all reported p values were two-sided. All analyses were conducted using SPSS software, v23.0 (SPSS Inc. Chicago, IL, USA), and R statistical package (v.4.0.2).

## Results

### Baseline characteristics

A total of 318 primary liver cancer patients undergoing hepatectomy were included in this study, of which 150 were in the ERAS group and 168 were in the non-ERAS group. In total, 246 individuals (77.4%) were males, 72 individuals(22.6%)were females, with an average age of 55.0 ± 10.5 years, and 106 individuals (33.3%) had a middle school education. The median BMI was 23.1 (IQR: 20.9–24.9), 45 individuals (14.2%) were chronic drinking, 79 individuals (24.8%) were chronic smokers, 45 individuals (14.2%) had comorbidities, 95 individuals (29.9%) chose laparoscopic surgery, the ASA score of I + II accounted most (70.8%), and most patients experienced single segmentectomy (43.7%). In addition, we compared the ERAS group and the non-ERAS group and found that the two groups had no statistical differences in baseline characteristics (all P values were > 0.05) (Table 1**)**.

**Table 1:** Perioperative baseline characteristics of patients with hepatocellular carcinoma in the ERAS and Non-ERAS groups

	All (N = 318)	ERSA (N = 150)	Non-ERSA (N = 168)	P
**Age at diagnosis (year)**	55.0 (10.5)	54.2 (10.8)	55.7 (10.1)	0.210
**Sex**				0.885
Female	72 (22.6%)	35 (23.3%)	37 (22.0%)	
Male	246 (77.4%)	115 (76.7%)	131 (78.0%)	
**BMI**	23.1 [20.9;24.9]	22.7 [20.6;24.6]	23.4 [21.3;25.3]	0.149
**Education level**				0.823
No school/primary education	79 (24.8%)	37 (24.7%)	42 (25.0%)	
Middle Education	106 (33.3%)	53 (35.3%)	53 (31.5%)	
High education	68 (21.4%)	29 (19.3%)	39 (23.2%)	
University education	65 (20.4%)	31 (20.7%)	34 (20.2%)	
**Comorbidity**				0.230
No	273 (85.8%)	133 (88.7%)	140 (83.3%)	
Yes	45 (14.2%)	17 (11.3%)	28 (16.7%)	
**Chronic smoking**				0.748
No	239 (75.2%)	111 (74.0%)	128 (76.2%)	
Yes	79 (24.8%)	39 (26.0%)	40 (23.8%)	
**Chronic drinking**				0.065
No	273 (85.8%)	135 (90.0%)	138 (82.1%)	
Yes	45 (14.2%)	15 (10.0%)	30 (17.9%)	
**ALT (U/L)**	26.0 [19.0;33.0]	25.0 [19.0;33.0]	27.0 [20.0;34.0]	0.264
**AST (U/L)**	24.0 [17.0;30.8]	23.0 [17.0;30.0]	25.0 [18.0;31.0]	0.375
**PLT (×109 L)**	194 [139;240]	193 [144;233]	196 [136;247]	0.546
**HB (g/L)**	138 [125;151]	138 [127;150]	138 [123;151]	0.587
**WB (×109 L)**	5.38 [4.38;6.64]	5.20 [4.26;6.51]	5.54 [4.67;6.83]	0.087
**Creatinine (umol/L)**	73.5 [63.0;82.8]	73.0 [62.0;81.0]	74.0 [64.0;84.0]	0.362
**ALB (g/L)**	41.2 [38.7;43.6]	41.5 [38.5;44.2]	41.0 [39.1;43.2]	0.310
**TBIL (umol/L)**	11.8 [8.53;15.9]	11.6 [8.60;14.8]	11.9 [8.50;17.2]	0.136
**ASA**:				0.185
I + II	225 (70.8%)	112 (74.7%)	113 (67.3%)	
III	93 (29.2%)	38 (25.3%)	55 (32.7%)	
**Surgical approach**				0.679
Laparoscopic technique	95 (29.9%)	47 (31.3%)	48 (28.6%)	
Open	223 (70.1%)	103 (68.7%)	120 (71.4%)	
**Extent of operation**				0.835
Single segmentectomy	139 (43.7%)	67 (44.7%)	72 (42.9%)	
Two combined segmentectomies	122 (38.4%)	55 (36.7%)	67 (39.9%)	
Major hepatectomy	57 (17.9%)	28 (18.7%)	29 (17.3%)	
**Surgical time (minute)**	270 [217;333]	274 [226;334]	270 [212;329]	0.287
**Blood volume intraoperative (ml)**	300 [100;500]	225 [100;5.00]	300 [100;500]	0.081

Continuous variables are presented as mean (SD, standard deviation) or median (IQR, interquartile range); the categorical variables are presented as numbers (%)

ERAS, enhanced recovery after surgery; BMI, body mass index; ALT, glutamic pyruvic transaminase; AST, glutamic oxaloacetic transaminase; PLT, platelet; HB, hemoglobin; WB; white blood cell; ALB, albumin; TBIL, total bilirubin; ASA, American Society of Anesthesiologists

### Outcome comparison between ERAS and non-ERAS group

Table 2 presented the short-term outcomes and postoperative complications between the ERAS and Non-ERAS groups. Overall, compared to patients in the non-ERAS group, patients who received the ERAS protocol had lower postoperative pain scores (postoperative day 2: [3 vs. 5, P < .001] and postoperative day 3: [2 vs. 4, P < .001]), faster gastrointestinal functional recovery (2 days vs. 3 days, P < .001) and shorter postoperative hospital stay (postoperative hospital stay within 6 days: 73.3% vs. 53.0%, P < .001) and fewer postoperative complications (any postoperative complication rate: 21.3% vs. 38.7%, P = .001). However, there was no significant difference in the total cost of treatment (median 81,601 yuan vs. 83,459 yuan, P = .516) and rehospitalization after discharge rate within 30 days (2.67% vs. 7.74%, P = .079) between the two groups.

**Table 2:** Comparison of short-term outcomes and postoperative complication between the ERAS and Non-ERAS groups

	All	ERSA	Non-ERSA	P
**POD1 pain score**	1.00 [1.00;2.00]	1.00 [1.00;2.00]	2.00 [1.00;2.00]	0.167
**POD2 pain score**	4.00 [3.00;5.00]	3.00 [2.00;4.00]	5.00 [4.00;6.00]	< 0.001
**POD3 pain score**	3.00 [2.00;4.00]	2.00 [1.00;3.00]	4.00 [3.00;5.00]	< 0.001
**Gastrointestinal function recovery days**	2.00 [2.00;3.00]	2.00 [1.00;2.00]	3.00 [2.00;4.00]	< 0.001
**Postoperative hospitalization days**	6.00 [5.00;7.00]	6.00 [5.00;7.00]	6.00 [5.00;8.00]	< 0.001
**Postoperative hospitalization days**				< 0.001
Six days or less	199 (62.6%)	110 (73.3%)	89 (53.0%)	
More than 6 days	119 (37.4%)	40 (26.7%)	79 (47.0%)	
**Total cost (RMB)**	82,638 [70,878;98,873]	81,601 [70,960;96,134]	83,459 [70,771;100,167]	0.516
**Rehospitalization after discharge (< 30 days)**				0.079
No	301 (94.7%)	146 (97.3%)	155 (92.3%)	
Yes	17 (5.35%)	4 (2.67%)	13 (7.74%)	
**Any postoperative complication**				0.001
No	221 (69.5%)	118 (78.7%)	103 (61.3%)	
Yes	97 (30.5%)	32 (21.3%)	65 (38.7%)	
**Wound infection**				0.321
No	279 (87.7%)	135 (90.0%)	144 (85.7%)	
Yes	39 (12.3%)	15 (10.0%)	24 (14.3%)	
**Deep vein thrombosis**				1.000
No	309 (97.2%)	146 (97.3%)	163 (97.0%)	
Yes	9 (2.83%)	4 (2.67%)	5 (2.98%)	
**Pleural effusion**				0.036
No	276 (86.8%)	137 (91.3%)	139 (82.7%)	
Yes	42 (13.2%)	13 (8.67%)	29 (17.3%)	
**Seroperitoneum**				0.166
No	281 (88.4%)	137 (91.3%)	144 (85.7%)	
Yes	37 (11.6%)	13 (8.67%)	24 (14.3%)	
**Biliary leakage**:				1.000
No	313 (98.4%)	148 (98.7%)	165 (98.2%)	
Yes	5 (1.57%)	2 (1.33%)	3 (1.79%)	
**Postoperative bleeding again**				0.453
No	311 (97.8%)	148 (98.7%)	163 (97.0%)	
Yes	7 (2.20%)	2 (1.33%)	5 (2.98%)	
**Nausea/ vomiting**				0.009
No	229 (72.0%)	119 (79.3%)	110 (65.5%)	
Yes	89 (28.0%)	31 (20.7%)	58 (34.5%)	
**Ventosity**				0.017
No	245 (77.0%)	125 (83.3%)	120 (71.4%)	
Yes	73 (23.0%)	25 (16.7%)	48 (28.6%)	

Continuous variables are presented as median (IQR, interquartile range); the categorical variables are presented as numbers (%)

ERAS, enhanced recovery after surgery; POD, postoperative day

### Risk factors for the incidence of postoperative prolonged hospital stay

To further analyze whether ERAS is an independent protective factor for a less prolonged postoperative hospital stay, we used logistic regression analysis to adjust for the effects of other confounding variables. First, we found that the median postoperative hospital stay in the ERAS group and the non-ERAS group was 6.00 (IQR: 5.00–7.00) and 6.00 (IQR: 5.00–8.00), respectively. We divided the postoperative hospital stay into two groups: less than or equal to 6 days and more than 6 days. We defined postoperative hospital stay greater than 6 days as the postoperative hospital stay delay group. Univariate logistic regression analysis showed that whether the patient had received ERAS protocol, surgical approach, the extent of the operation, blood volume intraoperative, and any postoperative complication were significant factors influencing postoperative hospitalization delay. Similarly, in the multivariate logistic regression analysis, we found that ERAS protocol, surgical approach, the extent of the operation, and blood volume intraoperative were independent factors for predicting postoperative hospital stay delay (Table 3).

**Table 3:** The risk factors for a postoperative prolonged hospital stay

	Postoperative hospitalization days of > 6 days			Risk factors of postoperative hospitalization days > 6 days		
				univariable	multivariable-1	multivariable-2
Variables	No (N = 199)	Yes (N = 119)	P	OR (95%CI)	OR (95%CI)	OR (95%CI)
**Group**			< 0.001			
ERAS	110 (55.3%)	40 (33.6%)		1 reference	1 reference	1 reference
Non-ERAS	89 (44.7%)	79 (66.4%)		2.44 (1.52–3.91, p < .001)	2.63 (1.57–4.43, p < .001)	2.64 (1.59–4.38, p < .001)
**Age at diagnosis**	54.8 (11.1)	55.2 (9.46)	0.726	1.00 (0.98–1.03, p = .735)		
**Sex**			0.689			
Female	47 (23.6%)	25 (21.0%)		1 reference		
Male	152 (76.4%)	94 (79.0%)		1.16 (0.67–2.01, p = .591)		
**BMI**	23.2 [20.9;24.8]	23.0 [20.9;25.5]	0.947	1.00 (0.93–1.08, p = .920)		
**Education level**			0.903			
No school/primary education	47 (23.6%)	32 (26.9%)		1 reference		
Middle Education	66 (33.2%)	40 (33.6%)		0.89 (0.49–1.62, p = .702)		
High education	44 (22.1%)	24 (20.2%)		0.80 (0.41–1.57, p = .517)		
University education	42 (21.1%)	23 (19.3%)		0.80 (0.41–1.59, p = .529)		
**Comorbidity**			0.91			
No	170 (85.4%)	103 (86.6%)		1 reference		
Yes	29 (14.6%)	16 (13.4%)		0.91 (0.47–1.76, p = .780)		
**Chronic smoking**			1			
No	150 (75.4%)	89 (74.8%)		1 reference		
Yes	49 (24.6%)	30 (25.2%)		1.03 (0.61–1.74, p = .907)		
**Chronic drinking**			0.376			
No	174 (87.4%)	99 (83.2%)				
Yes	25 (12.6%)	20 (16.8%)		1.41 (0.74–2.66, p = .295)		
**ALT (U/L)**	27.0 [20.0;33.0]	25.0 [18.0;33.5]	0.273	0.98 (0.96–1.01, p = .293)		
**AST (U/L)**	25.0 [17.0;31.0]	23.0 [18.0;29.0]	0.547	0.99 (0.96–1.02, p = .598)		
**PLT (×109 L)**	195 [138;240]	194 [140;242]	0.777	1.00 (1.00–1.00, p = .773)		
**HB (g/L)**	137 [125;151]	140 [124;150]	0.828	1.00 (0.99–1.01, p = .815)		
**WB (×109 L)**	5.43 [4.32;6.63]	5.23 [4.46;6.67]	0.766	0.97 (0.88–1.07, p = .545)		
**Creatinine (umol/L)**	74.0 [63.0;83.0]	73.0 [63.5;81.0]	0.535	0.99 (0.98–1.01, p = .410)		
**ALB (g/L)**	41.4 [39.0;43.8]	40.9 [38.7;43.2]	0.639	1.00 (0.95–1.06, p = .858)		
**TBIL (umol/L)**	12.0 [8.85;15.9]	11.6 [8.15;16.5]	0.665	1.00 (0.98–1.02, p = .976)		
**ASA**:			0.939			
I + II	140 (70.4%)	85 (71.4%)		1 reference		
III	59 (29.6%)	34 (28.6%)		0.95 (0.58–1.57, p = .838)		
**Surgical approach**			0.005			
Laparoscopic technique	71 (35.7%)	24 (20.2%)		1 reference	1 reference	1 reference
Open	128 (64.3%)	95 (79.8%)		2.20 (1.29–3.74, p = .004)	2.01 (1.12–3.59, p = .018)	2.01 (1.14–3.56, p = .016)
**Extent of operation**			< 0.001			
Single segmentectomy	97 (48.7%)	42 (35.3%)		1 reference	1 reference	1 reference
Two combined segmentectomies	81 (40.7%)	41 (34.5%)		1.17 (0.69–1.97, p = .557)	1.05 (0.61–1.83, p = .858)	1.05 (0.61–1.83, p = .857)
Major hepatectomy	21 (10.6%)	36 (30.3%)		3.96 (2.07–7.57, p < .001)	3.48 (1.71–7.08, p < .001)	3.48 (1.73–7.03, p < .001)
**Surgical time (minute)**	271 [222;330]	270 [210;335]	0.703	1.00 (1.00–1.00, p = .468)		
**Blood volume intraoperative (per 100ml)**	200 [100;500]	400 [200;600]	< 0.001	1.12 (1.06–1.19, p < .001)	1.08 (1.01–1.15, p = .016)	1.08 (1.02–1.15, p = .006)
**Any postoperative complication**			< 0.001			
No	153 (76.9%)	68 (57.1%)		1 reference	1 reference	
Yes	46 (23.1%)	51 (42.9%)		2.49 (1.53–4.07, p < .001)	1.01 (0.54–1.89, p = .974)	

Continuous variables are presented as mean (SD, standard deviation) or median (IQR, interquartile range); the categorical variables are presented as numbers (%)

ERAS, enhanced recovery after surgery; BMI, body mass index; ALT, glutamic pyruvic transaminase; AST, glutamic oxaloacetic transaminase; PLT, platelet; HB, hemoglobin; WB; white blood cell; ALB, albumin; TBIL, total bilirubin; ASA, American Society of Anesthesiologists; OR, odd ratio; CI, confidence interval

Multivariable-1 presents the results of including statistically significant variables in univariate analysis in logistics regression; multivariable-2 present the results of stepwise backward logistic regression

**Table 4:** The risk factors for any postoperative complication occurrence

	Any postoperative complication			Predictors of any postoperative complication occurrence		
				univariable	multivariable-1	multivariable-2
Variables	No (N = 221)	Yes (N = 97)	P	OR (95%CI)	OR (95%CI)	OR (95%CI)
**Group**			0.001			
ERAS	118 (53.4%)	32 (33%)		1 reference	1 reference	1 reference
Non-ERAS	103 (46.6%)	65 (67%)		2.33 (1.41–3.83, p < .001)	2.51 (1.30–4.84, p = .006)	2.58 (1.37–4.89, p = .004)
**Age at diagnosis**	53.9 (10.4)	57.5 (10.3)	0.004	1.04 (1.01–1.06, p = .004)	1.03 (1.00-1.07, p = .029)	1.04 (1.01–1.07, p = .014)
**Sex**			< 0.001			
Female	64 (29.0%)	8 (8.25%)		1 reference	1 reference	1 reference
Male	157 (71.0%)	89 (91.8%)		4.54 (2.08–9.89, p < .001)	3.91 (1.36–11.26, p = .011)	4.30 (1.65–11.23, p = .003)
**BMI**	23.0 [20.9;24.8]	23.4 [21.7;25.4]	0.175	1.06 (0.98–1.15, p = .133)		
**Education level**			0.526			
No school/primary education	58 (26.2%)	21 (21.6%)		1 reference		
Middle Education	69 (31.2%)	37 (38.1%)		1.48 (0.78–2.81, p = .229)		
High education	46 (20.8%)	22 (22.7%)		1.32 (0.65–2.69, p = .444)		
University education	48 (21.7%)	17 (17.5%)		0.98 (0.46–2.06, p = .954)		
**Comorbidity**			0.044			
No	196 (88.7%)	77 (79.4%)		1 reference	1 reference	
Yes	25 (11.3%)	20 (20.6%)		2.04 (1.07–3.88, p = .030)	1.40 (0.59–3.32, p = .439)	
**Chronic smoking**			0.498			
No	169 (76.5%)	70 (72.2%)		1 reference		
Yes	52 (23.5%)	27 (27.8%)		1.25 (0.73–2.16, p = .414)		
**Chronic drinking**			0.187			
No	194 (87.8%)	79 (81.4%)		1 reference		
Yes	27 (12.2%)	18 (18.6%)		1.64 (0.85–3.14, p = .138)		
**ALT (U/L)**	26.0 [20.0;33.0]	26.0 [19.0;33.0]	0.719	1.00 (0.97–1.03, p = .750)		
**AST (U/L)**	24.0 [17.0;30.0]	26.0 [19.0;31.0]	0.264	1.02 (0.99–1.05, p = .265)		
**PLT (×109 L)**	196 [142;241]	179 [133;232]	0.341	1.00 (0.99-1.00, p = .322)		
**HB (g/L)**	137 [125;151]	141 [128;150]	0.926	1.00 (0.99–1.01, p = .835)		
**WB (×109 L)**	5.38 [4.32;6.57]	5.44 [4.59;7.05]	0.316	1.06 (0.97–1.17, p = .208)		
**Creatinine (umol/L)**	72.0 [61.0;82.0]	75.0 [67.0;84.0]	0.013	1.02 (1.00-1.03, p = .028)	1.00 (0.98–1.03, p = .729)	
**ALB (g/L)**	41.4 [39.3;43.9]	40.4 [38.5;42.9]	0.037	0.95 (0.90–1.01, p = .077)		
**TBIL (umol/L)**	11.5 [8.70;15.5]	12.7 [8.50;16.6]	0.371	1.00 (0.98–1.02, p = .805)		
**ASA**:			0.617			
I + II	154 (69.7%)	71 (73.2%)		1 reference		
III	67 (30.3%)	26 (26.8%)		0.84 (0.49–1.43, p = .526)		
**Surgical approach**			< 0.001			
Laparoscopic technique	82 (37.1%)	13 (13.4%)		1 reference	1 reference	1 reference
Open	139 (62.9%)	84 (86.6%)		3.81 (2.00-7.26, p < .001)	3.07 (1.42–6.63, p = .004)	3.12 (1.45–6.72, p = .004)
**Extent of operation**			< 0.001			
Single segmentectomy	109 (49.3%)	30 (30.9%)		1 reference	1 reference	1 reference
Two combined segmentectomies	86 (38.9%)	36 (37.1%)		1.52 (0.87–2.67, p = .143)	1.43 (0.71–2.87, p = .312)	1.43 (0.71–2.84, p = .314)
Major hepatectomy	26 (11.8%)	31 (32.0%)		4.33 (2.24–8.38, p < .001)	2.74 (1.18–6.37, p = .019)	2.84 (1.23–6.56, p = .014)
**Surgical time (minute)**	267 [217;315]	285 [218;359]	0.053	1.00 (1.00-1.01, p = .004)	1.00 (1.00–1.00, p = .974)	
**Blood volume intraoperative (per 100ml)**	200 [100;400]	500 [300;1100]	< 0.001	1.40 (1.27–1.54, p < .001)	1.37 (1.23–1.52, p < .001)	1.36 (1.23–1.50, p < .001)

Continuous variables are presented as mean (SD, standard deviation) or median (IQR, interquartile range); the categorical variables are presented as numbers (%)

ERAS, enhanced recovery after surgery; BMI, body mass index; ALT, glutamic pyruvic transaminase; AST, glutamic oxaloacetic transaminase; PLT, platelet; HB, hemoglobin; WB; white blood cell; ALB, albumin; TBIL, total bilirubin; ASA, American Society of Anesthesiologists; OR, odd ratio; CI, confidence interval

Multivariable-1 presents the results of including statistically significant variables in univariate analysis in logistics regression; multivariable-2 present the results of stepwise backward logistic regression

### Risk factors for the incidence of postoperative complications

Univariate logistic regression analysis showed that whether patients received ERAS protocol, age at diagnosis, gender, comorbidity, preoperative serum creatinine level, surgical approach, the extent of the operation, surgical time, and intraoperative blood loss were statistical risk factors in predicting postoperative complications occurrence (Table 4). But in multivariate regression analysis, we just found that ERAS protocol, age at diagnosis, gender, surgical approach, the extent of the operation, and intraoperative blood loss were independent risk factors of postoperative complications.

## Discussion

In the present study, we found that the ERAS could reduce the risk of postoperative complication, accelerate gastrointestinal function recovery, reduce postoperative pain, and shorten the length of postoperative hospital stay for primary liver cancer patients, which are consistent with limited previous studies [7]. The ERAS group took a series of measures to reduce the occurrence of postoperative complications. The current 2022 released ERAS guideline for liver surgery showed that preoperative smoking and alcohol cessation, preoperative nutrition, wound catheter and transversus abdominis plane block, prophylactic nasogastric intubation, prophylactic abdominal drainage, postoperative artificial nutrition, and early oral intake, postoperative glycemic control, postoperative nausea and vomiting prevention, and fluid management have been demonstrated useful for liver surgery patients’ recovery [16].

Although our study shows that the median length of postoperative hospital stay is 6.00 (IQR: 5.00–7.00) and 6.00 (IQR: 5.00–8.00) for the ERAS group and the non-ERAS group, respectively, the proportion of patients with a postoperative hospital stay less than 6 days in the ERAS group was greater than that in the non-ERAS group, suggesting that ERAS has shortened the postoperative hospital stay of patients, which was also proved in multivariate logistic regression analysis. Our present results are consistent with Liang, et al. study [18] and they reported that the average length of hospital stay in the ERAS group was 6.2 days. High postoperative morbidity will lead to a prolonged length of hospital stay. ERAS can reduce postoperative complication occurrence; Therefore, it can also shorten the postoperative hospital stay of patients.

Although the clinical benefits of ERAS have been extensively studied, there are few studies on the cost-efficiency of ERAS in hepatectomy [19]. In this study, there is no statistical difference between the two groups. However, Joliat et al. [20] reported that the intraoperative cost of the ERAS group was higher than that of the non-ERAS group. Although ERAS will increase a certain cost, it can reduce postoperative complications, speed up gastrointestinal functional recovery, and shorten the length of postoperative hospital stay, all of which can reduce postoperative hospital costs. So, the hospital cost has reached the “break-even point”.

There was a significant difference in the pain score on the 2, and 3 days after the operation between the ERAS group and the non-ERAS group in the present study. At present, the postoperative ward analgesia in our center mainly adopts intravenous analgesia and local infiltration anesthesia of incision. Compared with the previous epidural analgesia, better management can avoid the occurrence of hypotension [16]. The ERAS group adopted not only multimodal analgesia but also preventive, timely, and on-demand analgesia. These measures can effectively reduce postoperative pain. Also, we found that the average pain score was not more than 4 in the ERAS groups. This shows that the postoperative moderate and severe pain has been effectively controlled in the ERAS group. However, in the results of Kapritsou et al. [21], there was no difference in pain score between the ERAS group and the non-ERAS group and suggested that the use of the behavioral observation scale and visual analog scale may be subjectively influenced by nurses. In the future, a variety of pain score scales and prospective experiments are needed to explore the relationship between ERAS and postoperative pain.

In this study, the ERAS group accelerated a median 1 day in gastrointestinal function recovery than the non-ERAS group. Chewing gum, oral laxatives, early feeding, and early mobilization can promote bowel movements and accelerate bowel movements. In addition, Simpson et al. [22] showed that postoperative opioid use is not only an important risk factor for postoperative nausea and vomiting but also related to the occurrence of postoperative intestinal obstruction. The analgesic drugs in ERAS are mainly non-steroidal anti-inflammatory and have fewer opiates, which can not only reduce postoperative nausea and vomiting but also accelerate gastrointestinal functional recovery.

A prior study showed that is no difference in the admission rate (<30 days) between the ERAS group and the non-ERAS group [5]. In our present study, although ERAS reduced the rate of readmission <30 days (2.67% vs. 7.74%), we also found that there is no statistical significance between the ERAS group and the non-ERAS group (p = .079). The main measures of ERAS are used in the perioperative period, and they are not aimed at the continuation of care after discharge.

Our study is not devoid of limitations. In this study, we use the method of prospectively and retrospectively collecting no-ERAS and ERAS group data, respectively, which leads to not completely randomized grouping. Besides, this study only collected data from a single center, which has limitations. In the future, prospective and multicenter experiments are needed to verify the effect of ERAS after hepatectomy for primary liver cancer patients.

## Conclusion

In the present study, we found that ERAS can accelerate gastrointestinal function recovery, reduce postoperative pain and postoperative complications, and shorten the length of postoperative hospital stay for primary liver cancer patients treated with hepatectomy. In multivariate regression analysis, we found that patients who implemented the ERAS protocol had a lower risk of postoperative complications and prolonged hospital stay, and these were statistically significant factors. At the same time, we need to point out that the occurrence of postoperative complications in addition to whether the patient implements the ERAS protocol, the difficulty of the hepatectomy itself is an important independent factor. Overall, the application of ERAS in hepatectomy for patients with liver cancer is safe and effective.

## Electronic supplementary material

Below is the link to the electronic supplementary material.

Supplementary Table 1: Perioperative procedures

## Data Availability

The datasets used and/or analyzed during the current study are available from the corresponding author upon reasonable request.

## References

[1] Xia C, Dong X, Li H, Cao M, Sun D, He S, Yang F, Yan X, Zhang S, Li N (2022). Cancer statistics in China and United States, 2022: profiles, trends, and determinants. Chin Med J (Engl).

[2] Siegel RL, Miller KD, Fuchs HE, Jemal A (2022). Cancer statistics, 2022. CA Cancer J Clin.

[3] Forner A, Reig M, Bruix J (2018). Hepatocellular carcinoma. The Lancet.

[4] Komorowski AL, Mituś JW, Wysocki WM, Bała MM (2017). Laparoscopic and open liver resection – a literature review with meta-analysis. Archives of Medical Science.

[5] He F, Lin X, Xie F, Huang Y, Yuan R (2015). The effect of enhanced recovery program for patients undergoing partial laparoscopic hepatectomy of liver cancer. Clin Transl Oncol.

[6] Berardi G, Morise Z, Sposito C, Igarashi K, Panetta V, Simonelli I, Kim S, Goh BKP, Kubo S, Tanaka S (2020). Development of a nomogram to predict outcome after liver resection for hepatocellular carcinoma in child-pugh B cirrhosis. J Hepatol.

[7] Feng J, Li K, Xu R, Feng H, Han Q, Ye H, Li F (2022). Association between compliance with enhanced recovery after surgery (ERAS) protocols and postoperative outcome in patients with primary liver cancer undergoing hepatic resection. J Cancer Res Clin Oncol.

[8] Li J, Huang L, Yan J, Qiu M, Yan Y (2018). Liver resection for hepatocellular carcinoma: personal experiences in a series of 1330 consecutive cases in China. ANZ J Surg.

[9] Kehlet H (1997). Multimodal approach to control postoperative pathophysiology and rehabilitation. Br J Anaesth.

[10] Ripolles-Melchor J, Ramirez-Rodriguez JM, Casans-Frances R, Aldecoa C, Abad-Motos A, Logrono-Egea M, Garcia-Erce JA, Camps-Cervantes A, Ferrando-Ortola C, Suarez de la Rica A (2019). Association between Use of enhanced recovery after surgery protocol and postoperative complications in colorectal surgery: the postoperative outcomes within enhanced recovery after surgery protocol (POWER) study. JAMA Surg.

[11] Pruthi RS, Nielsen M, Smith A, Nix J, Schultz H, Wallen EM (2010). Fast track program in patients undergoing Radical Cystectomy: results in 362 consecutive patients. J Am Coll Surg.

[12] Carter-Brooks CM, Du AL, Ruppert KM, Romanova AL, Zyczynski HM. Implementation of a urogynecology-specific enhanced recovery after surgery (ERAS) pathway. *American Journal of Obstetrics and Gynecology* 2018, 219(5):495.e491-495.e410. 10.1016/j.ajog.2018.06.009 PMC7212495 29913175

[13] Wainwright TW, Gill M, McDonald DA, Middleton RG, Reed M, Sahota O, Yates P, Ljungqvist O (2019). Consensus statement for perioperative care in total hip replacement and total knee replacement surgery: enhanced recovery after surgery (ERAS®) Society recommendations. Acta Orthop.

[14] Engelman DT, Ben Ali W, Williams JB, Perrault LP, Reddy VS, Arora RC, Roselli EE, Khoynezhad A, Gerdisch M, Levy JH (2019). Guidelines for Perioperative Care in Cardiac surgery: enhanced recovery after surgery Society Recommendations. JAMA Surg.

[15] Rouxel P, Beloeil H (2019). Enhanced recovery after hepatectomy: a systematic review. Anaesth Crit care pain Med.

[16] Joliat GR, Kobayashi K, Hasegawa K, Thomson JE, Padbury R, Scott M, Brustia R, Scatton O, Tran Cao HS, Vauthey JN (2023). Guidelines for Perioperative Care for Liver surgery: enhanced recovery after surgery (ERAS) Society Recommendations 2022. World J Surg.

[17] Melloul E, Hubner M, Scott M, Snowden C, Prentis J, Dejong CH, Garden OJ, Farges O, Kokudo N, Vauthey JN (2016). Guidelines for Perioperative Care for Liver surgery: enhanced recovery after surgery (ERAS) Society Recommendations. World J Surg.

[18] Liang X, Ying H, Wang H, Xu H, Yu H, Cai L, Wang Y, Tong Y, Ji L, Luo R et al. Enhanced recovery program Versus Traditional Care in Laparoscopic Hepatectomy. Medicine 2016, 95(8). 10.1097/MD.0000000000002835 PMC4779010 26937913

[19] Joliat GR, Hubner M, Roulin D, Demartines N (2020). Cost analysis of enhanced Recovery Programs in Colorectal, pancreatic, and hepatic surgery: a systematic review. World J Surg.

[20] Joliat GR, Labgaa I, Hubner M, Blanc C, Griesser AC, Schafer M, Demartines N (2016). Cost-benefit analysis of the implementation of an enhanced recovery program in liver surgery. World J Surg.

[21] Kapritsou M, Konstantinou EA, Korkolis DP, Kalafati M, Kaklamanos I, Giannakopoulou M (2018). Postoperative stress and pain response applying fast-track protocol in patients undergoing hepatectomy. J Perioper Pract.

[22] Simpson JC, Bao X, Agarwala A (2019). Pain Management in enhanced recovery after surgery (ERAS) protocols. Clin Colon Rectal Surg.

