# Supplementary material for: Enhanced recovery after surgery in primary liver cancer patients undergoing hepatectomy: experience from a large tertiary hospital in China
Source: BMC Surg. 2023 Jun 29;23:185. doi: 10.1186/s12893-023-02040-4 (PMC10311806; doi:10.1186/s12893-023-02040-4)
Supplement: Supplementary file 1 — Supplementary Table 1: Perioperative procedures [file 12893_2023_2040_MOESM1_ESM.docx]

| **Supplementary Table 1** Perioperative procedures | |
| --- | --- |
| **ERAS Group** | **No-ERAS Group** |
| **Preoperative** |  |
| Adopt multi-mode health education through education manuals, videos, WeChat public accounts, etc. | Routine oral education |
| Preoperative psychological comfort, appropriate physical exercise, quitting smoking and drinking, and viral hepatitis patients continue to give antiviral treatment. | If patients with viral hepatitis continue to be given antiviral therapy. |
| The attending physician and nutritionist carried out an individualized nutritional risk assessment for the patients, carried out individualized enteral nutrition intervention for the patients with malnutrition, and evaluated the nutritional status of the patients according to the dynamic condition, and then adjusted the enteral supplementation at least 1-2 weeks prior to surgery. | Patients were routinely assessed for nutritional risk, and patients with malnutrition were given a nutritional intervention by intravenous infusion of nutrient solution. |
| Long-acting sedative and anti-anxiety drugs should be avoided before the operation. | No special requirements. |
| There was no conventional mechanical enema before the operation, no gastric tube was placed before the operation, and the gastric tube was removed as soon as possible after the operation if the condition needed to be placed. | Preoperative routine use of 130 ml glycerol enema, preoperative routine placement of gastric tube 30 minutes. |
| Blood glucose was monitored 2 hours before surgery and 350 ml of carbohydrate (10 % glucose solution) was taken orally. | Preoperative fasting for 12 hours, no drinking for 4 hours. |
| Intravenous antibiotics were routinely used half an hour to an hour before skin incision. | Not used routinely. |
| Preoperatively, antithrombotic drugs and mechanical prevention and treatment are determined according to the risk of thrombosis. The drugs are mainly low molecular weight heparin. | No special requirements. |
| **Intraoperative** |  |
| Anesthesia induction and general anesthesia were performed with conventional anesthetics. | Anesthesia induction and general anesthesia were performed with conventional anesthetics. |
| A medical warm blanket was used to maintain the patient's body temperature during the operation. | During the operation, the patient's body temperature was maintained by thick quilts and heaters. |
| Select the appropriate patients for laparoscopic surgery, less abdominal drainage tube. | Appropriate patients were selected for laparoscopic surgery, and abdominal drainage tubes were routinely placed. |
| **Postoperative** |  |
| Take preventive, timely, multimodal analgesia. Intravenous analgesia and local infiltration anesthesia were routinely used in the postoperative ward. Intravenous analgesic drugs were mainly sufentanil, butorphanol, oxycodone, etc., and local infiltration anesthesia with incision ropivacaine was given. The patients were scored for pain, and intravenous analgesics and topical analgesic patches were given on time and on demand. | On-demand single-mode analgesia. |
| The combination of metoclopramide and ondansetron is used to prevent postoperative nausea and vomiting. | A single drug is used to prevent postoperative nausea and vomiting. |
| The gastric tube was removed as soon as possible after the operation, the urinary tube was removed 6 hours after anesthesia, and the abdominal drainage tube was removed without active bleeding. | After anesthesia, the gastric tube was removed after awakening, the urinary tube was removed after getting out of bed, and the abdominal drainage tube was removed after the daily drainage volume < 50 ml. |
| After returning to the ward after surgery, ankle pump exercise was performed in bed to prevent thrombosis, straight leg elevation, muscle contraction exercise, and family members assisted passive exercise. The first day after the operation: at least 4 times a day to get out of bed; the second day after surgery: walking 50-100 meters; the third day after the operation: free movement. | Three days after the operation, bed rest was the main activity, voluntary activity, or passive activity with the help of nursing staff. |
| Drinking water began 6 hours after surgery and gradually transitioned to a normal diet after surgery. One day after surgery, it is recommended to drink 100-200ml porridge or fish soup; two days after surgery, eat a semi-liquid diet, such as egg soup, up to 500 ml; after 3 days, a normal diet; chewing gum at ordinary times. | Restoration of intestinal peristalsis and anal exhaust after diet. |
